# Thoracic Outlet Syndrome after Minimally Invasive Repair of Pectus Excavatum in a 15-Year-Old Boy: A Case Report

**DOI:** 10.1055/s-0042-1748316

**Published:** 2022-07-19

**Authors:** Sara Fernandes, Carolina Soares-Aquino, Joana Monteiro, Norberto Estevinho, Mariana Borges-Dias

**Affiliations:** 1 Department of Pediatric Surgery, Centro Hospitalar Universitário de São João, Alameda Prof. Hernani Monteiro, Porto, Porto, Portugal

**Keywords:** Nuss procedure, pectus excavatum, thoracic outlet

## Abstract

Nuss procedure has become the treatment of choice in pectus excavatum mainly because of the excellent functional and cosmetic results. Despite the good results, several complications have been reported. The aim of this study is to describe a case of thoracic outlet syndrome (TOS) after Nuss procedure and review the management of such rare complication. A 15-year-old boy otherwise healthy was submitted to Nuss procedure, with no perioperative complications. Two-weeks later, the patient complained of right-hand paresthesia, progressive weakness of the right arm and coldness. After imaging and electromyography, TOS diagnosis was established. Removal of the bar was proposed but refused by the patient. Conservative management with rehabilitation exercising and nerve nourishing was initiated. At 7 months, the patient recovered arm and hand function. Abrupt structural changes of thoracic cavity with marked elevation of the upper chest induce nerve and vascular compression arousing a TOS and should be acknowledged as one potential complication of Nuss procedure. Conservative management can be an alternative treatment to bar removal, showing good results on functional recovery in early stages of compression.

## Introduction

Pectus excavatum (PE) is the most common chest wall deformity affecting ∼1 to 8 per 1,000 live births. 1 It is characterized by a dorsal deviation of the sternum and costal cartilages with variable degrees of cardiopulmonary physiological impairment due to mechanical compression. 2 3 Affected children often complain with chest pain, fatigue, and exercise intolerance. Besides, problems with cosmesis and psychological issues are also reported and bear a significant impact on patient's quality of life. 2 Several surgical procedures are used to correct this deformity. Nuss procedure is a minimally invasive technique that enables correction of the thoracic deformity by elevation of the concave part of the thorax, maintaining this correction with metal bars placed underneath the elevated cartilages and sternum. 4 Although patient's satisfaction improve significantly after Nuss procedure, associated complications are well recognized and potentially life threatening. 4 5

Herein, we describe an unusual complication after PE correction by Nuss technique related to the marked structural change of the thoracic cavity induced by the elevation of the depressed thorax.

## Case Report

**Fig. 1:**
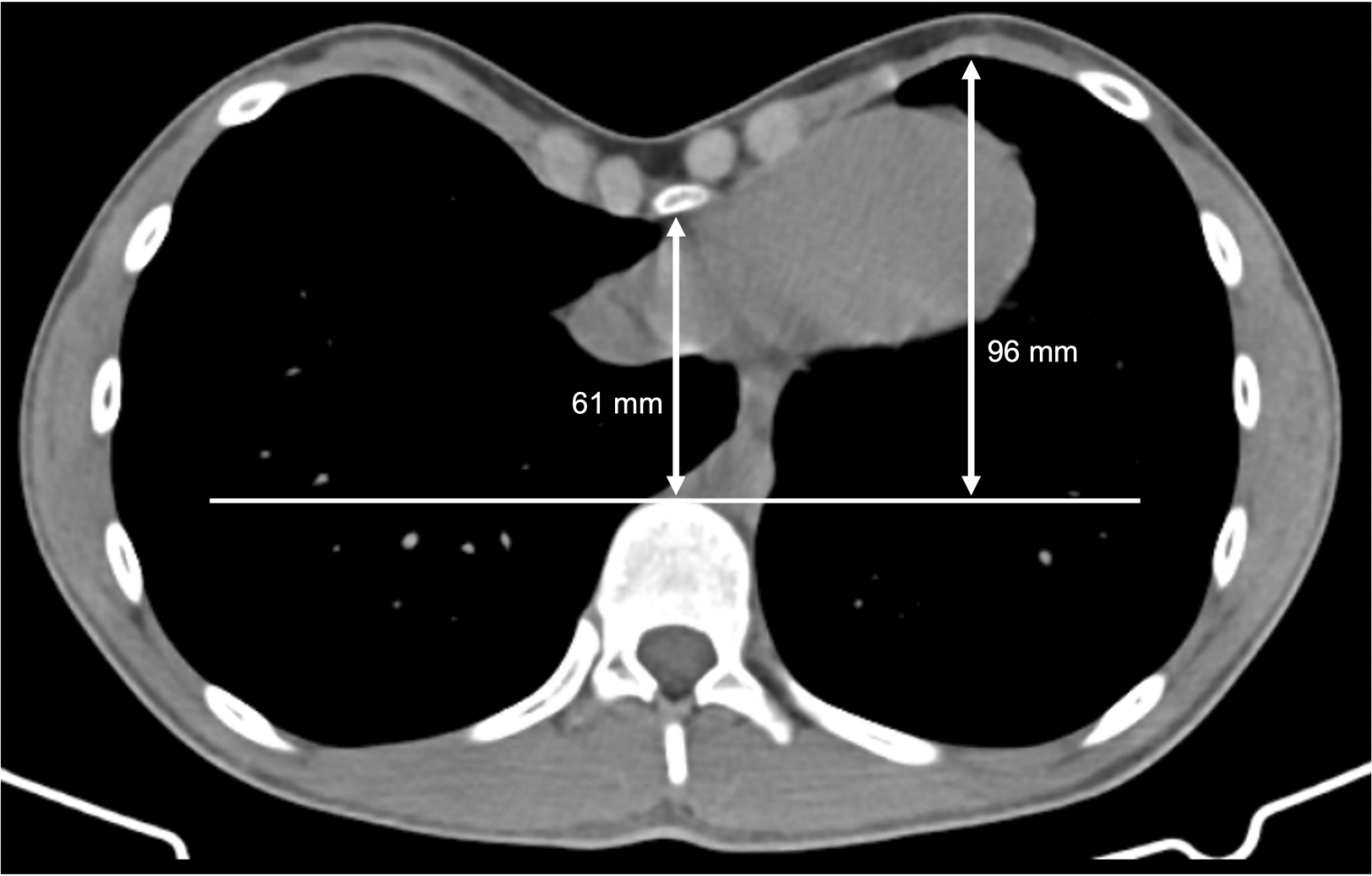
Preoperative thoracic computed tomography scan: axial image with correction index measures.

A 15-year-old boy was referred to pediatric surgery consultation with symptomatic chest wall deformity. A severe PE deformity with a Haller index of 4 (correction index of 36.5%) on the thoracic computed tomography was documented ( Fig. 1 ). Surgical correction was performed as described by Nuss et al 4 and one stainless-steel bar was inserted at the fourth intercostal space. The procedure underwent uneventfully and the bar stabilizer was placed on the right side and was tied with 1/0 polypropylene suture. Immediate postoperative period elapsed without complications and the patient was discharged at day 5, with a normal X-ray. Two weeks later, the patient started to complain of right-hand paresthesia, progressive weakness, and right arm coldness. On physical examination, loss of right-sided radial pulse was seen with elevated arms and improvement of symptoms was observed with arm adduction and lying position. Roos' test was performed and found to be positive.

Arterial Doppler ultrasonography confirmed compression of subclavian artery on costoclavicular space only in orthostatic position. Electromyogram findings corroborated the radicular lesion of C8-T1.

After establishing thoracic outlet syndrome (TOS) diagnosis, removal of the thoracic bar was proposed. Since both the patient and the parents were highly satisfied with the esthetic result of the procedure, they refused to undo the procedure and asked for other therapeutic solutions. Conservative management was offered, and the patient underwent rehabilitation exercises and nerve nourishing therapy was given with supplementation with vitamin B1. The rehabilitation program included progressive strengthening of the shoulder girdle to achieve normal position of the scapula and humeral head control and escalating ranges of arm abduction. Cervical mobilization for scalene triangle release and sensory re-education of the right upper limb were also performed.

Progressive recovery of muscle function was observed and the patient became asymptomatic. He recovered total arm and hand muscular function at 7-month follow-up.

## Discussion

Described by Nuss in 1987, minimally invasive technique for PE correction was presented as an appealing alternative to the standard open repair. 4 6 The Nuss procedure consists in the placement of one or more metallic bars in a retrosternal position that causes the elevation of the deepest point of depression of the deformity, under thoracoscopic control. 6 By taking advantage of the flexibility of the thorax in young subjects, the placement of these bars improves functional consequences of the deformity while having excellent cosmetic outcome. Nonetheless, several-associated complications have been described. 7 The most common complication is bar displacement (9.2%) followed by pneumothorax requiring chest tube drainage (4.8%). 7 Between the rarest complications, cardiac injury, sternal erosion, and pseudoaneurysm of the anterior thoracic artery are the most frequently reported.

TOS is also a rare encountered complication of Nuss procedure. 8 9 10 11 The morphological change of the thoracic wall after Nuss procedure can affect regional nerves and vessels. 11 The elevation of the upper chest conditioned by the sternal lifting after bar placement may narrow the space between the first rib and the clavicle, eventually causing compression of the neurovascular. Kim et al confirmed this structural changes in their study, by measuring costoclavicular spaces in PE patients and control groups. 12 They found that patients with PE originally have a narrower costoclavicular space than the normal controls, and this space narrow even more after surgery.

TOS is defined by a symptom complex that comprises pain, paresthesia, weakness, and discomfort of the upper limb aggravated by some movements. 13 This syndrome develops when the neurovascular bundle comprising brachial plexus, subclavian artery, and vein are compressed anywhere along their way from the thorax to the upper limb. Three sites of compression are possible: beneath the anterior scalene muscle, in the costoclavicular space, or beneath the pectoralis minor muscle. 11 13 Several causes of compression are known. Soft tissue and osseous alterations are the most frequent culprits attributed to TOS. 13 14

Herein, we report a case of a rare complication after Nuss procedure in an adolescent because of the acute structural changes in the thoracic wall. As previously described, the osseous shape alteration after the procedure probably cause narrowing of the space between the first rib and the clavicle. This leads to compression of the subclavian artery causing transient symptoms of acute ischemia, reverted by alteration of the arm positioning. Others have also described this rare complication ( Table 1 ).

**Table 1:** Review of literature on TOS post-Nuss procedure

Author (year of publication)	Patient characteristics	Time until symptoms	Symptoms	Imagiological confirmation	Treatment	Outcome
Kiliç et al (2013) 8	Male, 22 y	1 mo	Cold Weakness Pain	Doppler US Vascular MRI	Surgical resection of the first rib	Full improvement
Zhang et al (2018) 9	Male, 27 y	2 mo	Weakness Pain Numbness	EMG CT arteriogram	Conservative (nerve nourishing, exercises)	Muscular strength improvement Asymptomatic
Lee et al (2011) 10	Male, 13 y	3 d	Paresthesia Hypoesthesia Cold Claw hand deformity	EMG	Removal of upper thoracic bar	Recovery of sensory function No improvement of claw hand
Nagasao et al (2017) 11	Cohort ( *n* = 85) 7 males; 7 females	–	Pain Lassitude	No	None	Progressive improvement

Abbreviations: CT, computed tomography; EMG, electromyography; MRI, magnetic resonance imaging; TOS, thoracic outlet syndrome; US, ultrasound.

TOS treatment choice is highly controversial and depends whether there is a predominant vascular or nerve compression and the duration and evolution of symptoms. 13 14 Vascular compression frequently warrants surgical treatment and in neurogenic TOS with persistence of symptoms, muscle atrophy or progressive deficits surgical intervention should also be considered. 14 Anyway, conservative management is almost universally accepted as the first step in the treatment of TOS. 13 15 In TOS post-Nuss procedure, it is consensual that the removal of the bar and reversal of the induced thoracic structural change should be offered. However, the cosmetic outcomes of Nuss procedure are frequently much more attractive than is the amelioration of TOS symptoms. In most of the existent reports on TOS post-Nuss, patients are usually reluctant to consent with removal of the bar, at least initially. 8 9 10 This was the reason why Kiliç et al. offered other surgical procedure, thus preserving cosmetic results and patient satisfaction. The only patient between the reported TOS post-Nuss cases ( Table 1 ) that finally consented the removal of the bar did not experienced improvement the hand deformity probably because of irreversible nerve damage. 10 As in our case, Zhang et al showed good results with conservative measures, allying the advantages of precluding another surgical intervention and preserving the cosmetic appearance.

According to Nagasao et al, 11 female patients and those with severe forms of PE are at a higher risk of developing TOS after PE correction. In this work, it is also stated that a superior placement of the thoracic bars leads to a higher elevation of the sternum, augmenting the risk of neurovascular bundle's compression. Hence, surgeons must be aware of this when operating higher risk patients since some actions might prevent this complication. Lower bar placement should be attempted, provided that equally optimal correction is achieved. If a higher location is indispensable to achieve optimal results, a higher risk of TOS must be acknowledged and careful monitoring should be pursued, ensuring an early diagnosis and treatment.

## Conclusion

Being Nuss technique the actual state of the art in the treatment of PE, surgeons must be aware of this complication to its early recognition and treatment, preventing irreversible nerve damage. Although the existing literature is scarce, conservative treatment showed good outcomes while maintaining the final aspect of the thoracic wall. In the authors' opinion, it should be considered the first line of treatment in TOS post-Nuss. Progressive neuromuscular deficits or intractable symptoms should incite bar removal.

## Notes

Importance and New Insight for a Pediatric Surgeon

- to recognize a rare complication of Nuss procedure such as thoracic outlet syndrome.
- to acknowledge that conservative treatment is feasible and effective.
